# Physeal histological morphology after thermal epiphysiodesis using radiofrequency ablation

**DOI:** 10.1007/s10195-016-0430-y

**Published:** 2016-10-05

**Authors:** Juan Manuel Shiguetomi-Medina, B. Møller-Madsen, O. Rahbek

**Affiliations:** 1 Orthopaedics Research Laboratory, Aarhus University Hospital, Noerrebrogade 44, 8000 Aarhus C, Denmark; 2 Department of Children’s Orthopaedics, Aarhus University Hospital, Noerrebrogade 44, 8000 Aarhus C, Denmark

**Keywords:** Radiofrequency ablation, Epiphysiodesis, Physeal morphology, Histology

## Abstract

**Background:**

Several treatments have been described for leg length discrepancy. Epiphysiodesis is the most commonly used because of its effectiveness. Thermal epiphysiodesis using radiofrequency ablation (RFA) alters the growth plate morphology without damaging the adjacent articular cartilage; it is a minimally invasive method that has shown excellent results in animal models. This study describes the macro and micro morphology after the procedure.

**Materials and methods:**

Epiphysiodesis using RFA was performed in vivo for 8 min (92–98 °C) at two ablation sites (medial and lateral) in one randomly-selected tibia in eight growing pigs. The contralateral tibia was used as control. After 12 weeks, the pigs were killed and the tibiae harvested. The specimens were studied macroscopically and histology samples were obtained. Physeal morphology, thickness and characteristics were then described.

**Results:**

Macroscopically, the articular cartilage was normal in all the treated tibiae. Microscopically, the physis was detected as a discontinuous line on the treated tibiae while it was continuous in all controls. In the control specimens, the mean thickness of the physis was 625 µm (606–639, SD = 14). All the physeal layers were organized. In the ablated specimens, disorganized layers in a heterogeneous line were observed. Bone bridges were identified at the ablation sites. The central part of the physis looked normal. Next to the bone bridge, the physis was thicker and presented fibrosis. The mean thickness was 820 µm (628–949, SD = 130). No abnormalities in the articular cartilage were observed.

**Conclusions:**

Thermal epiphysiodesis with RFA disrupts the physeal morphology and causes the formation of bone bridges at the ablation sites. This procedure does not damage the adjacent articular cartilage. The damaged tissue, next to the bone bridges, is characterized by disorganization and fibrosis.

## Introduction

**Fig. 1:**
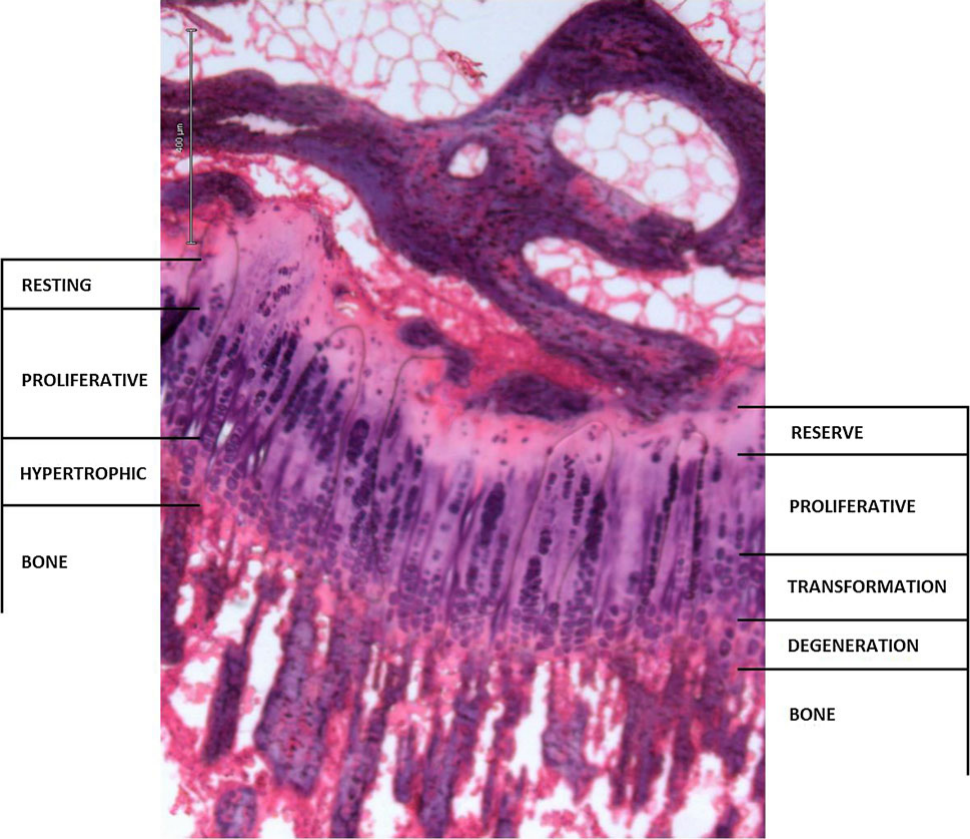
Growth plate zones, ×10. It is possible to identify the functional classification suggested by Delgado-Martos et al. (*right*) [2], and the histological classification suggested by Marino (*left*) [3]

The growth plate is the most fragile area of growing bones. Due to its poor ability to regenerate itself after injury, the growth plate injury site often has structural disorganization with formation of vertical septa and bone bridges. Bone bridge formation may be due to destruction of the epiphyseal circulation, but the mechanisms of bone bridge formation have not yet been clarified [1, 2]. Formation of bone bridges could be considered as a therapeutic goal after epiphysiodesis. Since its first description by Phemister in 1933, epiphysiodesis has become a common treatment for correction of deformities in growing children. Several techniques are currently used and all the non-reversible ones are used to obtain a permanent closure of the growth plate [3]. Thermal epiphysiodesis using radiofrequency ablation (RFA) has been reported as a successful treatment that can arrest growth in rabbits [4–6]. A temperature between 50 and 60 °C applied for 3 min to a cartilage tissue is enough to cause thermal coagulation [7]. Studies made in rabbits describe the histological characteristics induced after epiphysiodesis. In addition, this method has been proved to be successful in a porcine model, as it stopped growth when applied to a bone physis [8]. However, the effects of RFA epiphysiodesis in large species have not yet been described, to our knowledge. The histology of two reversible methods has been studied previously [3]. Micro-characteristics are important, sometimes crucial, to understanding disease and treatment. These studies are both useful and practical because they provide evidence and make concepts clear. The growth plate (epiphyseal plate or physis) is a highly specialized structure that develops from the cartilaginous-orientated mesenchymal cells. It is a sandwich-like multilayer structure divided into four well defined zones: reserve, proliferative, transformation and degeneration (Fig. 1). It is composed of hyaline cartilage tissue with a gelatinous texture and, when normal, is distributed in isogenic columns [9]. Resting cartilage cells lying within the reserve zone are formed by small, uniform, compactly located chondrocytes. The direct continuation of the reserve zone is the second layer, known as the proliferative zone. Its chondrocytes are flat and well divided into longitudinal columns. Below the proliferative zone is another layer known as the transformation zone, divided into upper and lower hypertrophic zones and farther located degeneration zone. Compared to the other zones, the chondrocytes of the transformation zone are relatively larger. Closer to the primary spongiosa zone, the number of cells with features of degeneration increases, chondrocytes lose intercellular junctions and are located in special vesicles. Finally, the last zone is adjacent to the primary spongiosa zone that provides the origin for the secondary spongiosa zone [10]. Histologically, it is easier to divide the growth plate into three distinct zones: resting, proliferative and hypertrophic (Fig. 1). The resting zone cells divide slowly and result in rapidly dividing proliferative cells which are arranged in orderly columns along the long axis of the bone. Proliferative cells terminally differentiate into hypertrophic chondrocytes which ultimately undergo apoptosis and contribute to ossification and new bone growth [11]. The aim of this study is to describe the growth plate morphology after epiphysiodesis using RFA in a porcine animal model.

## Materials and methods

One tibia was selected and treated using RFA in eight healthy, skeletally immature, 10-week-old female Danish Landrace pigs, average 38 kg in weight (36.5–40.8). These were followed for 12 weeks. After this period, the animals were killed and both tibiae harvested for analysis. Untreated tibiae served as control specimens

### Anesthesia and medication

All the procedures were done under general anesthesia. For premedication the animals received an intramuscular injection of ketaminol (5 mg/kg, S-ketamine, Pfizer, Berlin, Germany) and midazolam (0.5 mg/kg, Hypnomidate, Janssen-Cilag, Beerse, Belgium). Anesthesia was maintained with an intravenous infusion of propofol (5 mg/kg/h, Fresenius Kabi AB, Uppsala, Sweden) and fentanyl (0.025 mg/kg/h, Haldid, Janssen-Cilag). Before the ablation, the animals received one prophylactic intramuscular penicillin dose (1 ml/10 kg, procaine penicillin G 150 000 IU/1 ml), and then repeatedly daily for 3 days. The animals were given intramuscular flunixin (2.2 mg/kg, Finadyne Vet, Schering-Plough, Skovlunde, Denmark) for 3 days after the procedure.

### RFA epiphysiodesis

**Fig. 2:**
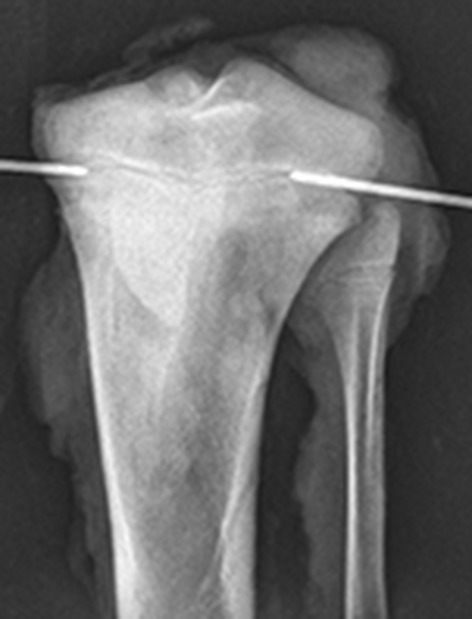
Epiphysiodesis procedure. The RFA probe is inserted laterally and medially into the proximal tibia growth plate

The RFA procedures were done at the experimental surgical research facilities of the Department of Clinical Medicine, Aarhus University Hospital Skejby. Using sterile surgical technique, a 14 G bone biopsy penetration set was used (Bonopty^®^ Bone Biopsy System, AprioMed AB, Uppsala, Sweden) under fluoroscopic guidance to reach the growth plate 90° from the vertical plane. The radiofrequency probe was then inserted 1 cm into the growth plate and the ablation was performed for 8 min (Fig. 2), maintaining the temperature of the probe between 92 and 98 °C. The procedure was done in the lateral and medial part of the growth plate.

### Follow-up

Animals were housed in individual pens at Aarhus University Påskehøjgaard Research Center, Trige, Denmark. They were fed standard Danish diet to which they had free access together with water. Animal-care staff took daily care of the animals.

### Euthanasia

At the end of the study, under general anesthesia, the animals received a lethal intravenous injection of pentobarbital 0.5 ml/kg.

### Sampling

**Fig. 3:**
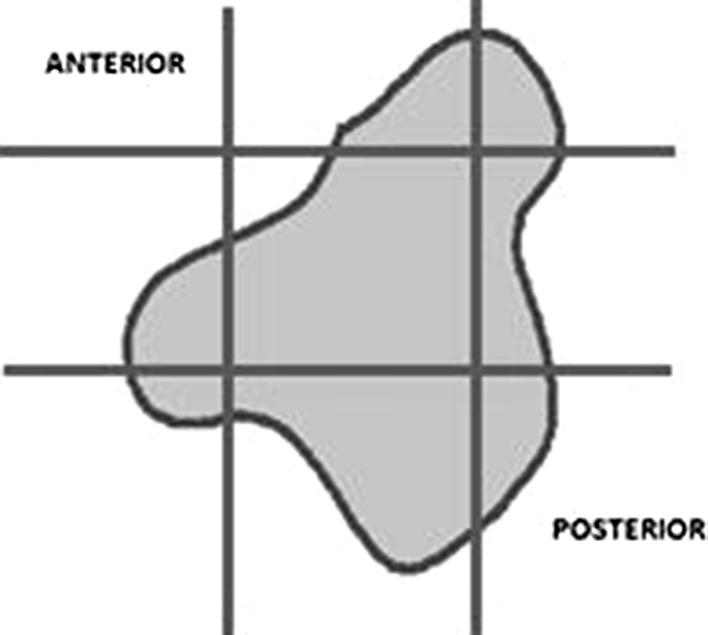
Transversal view of a proximal tibia epiphysis scheme. The *blue lines* represent the cuts that were made to get the samples for histology (color figure online)

Each tibia was cut 15 cm below the proximal articular cartilage. Each epiphysis was randomly rotated over its vertical axis and two equidistant coronal parallel cuts were made, followed by two parallel equidistant cuts perpendicular to the first two (Fig. 3). Eight or nine tissue samples were obtained from each epiphysis.

### Histology

**Fig. 4:**
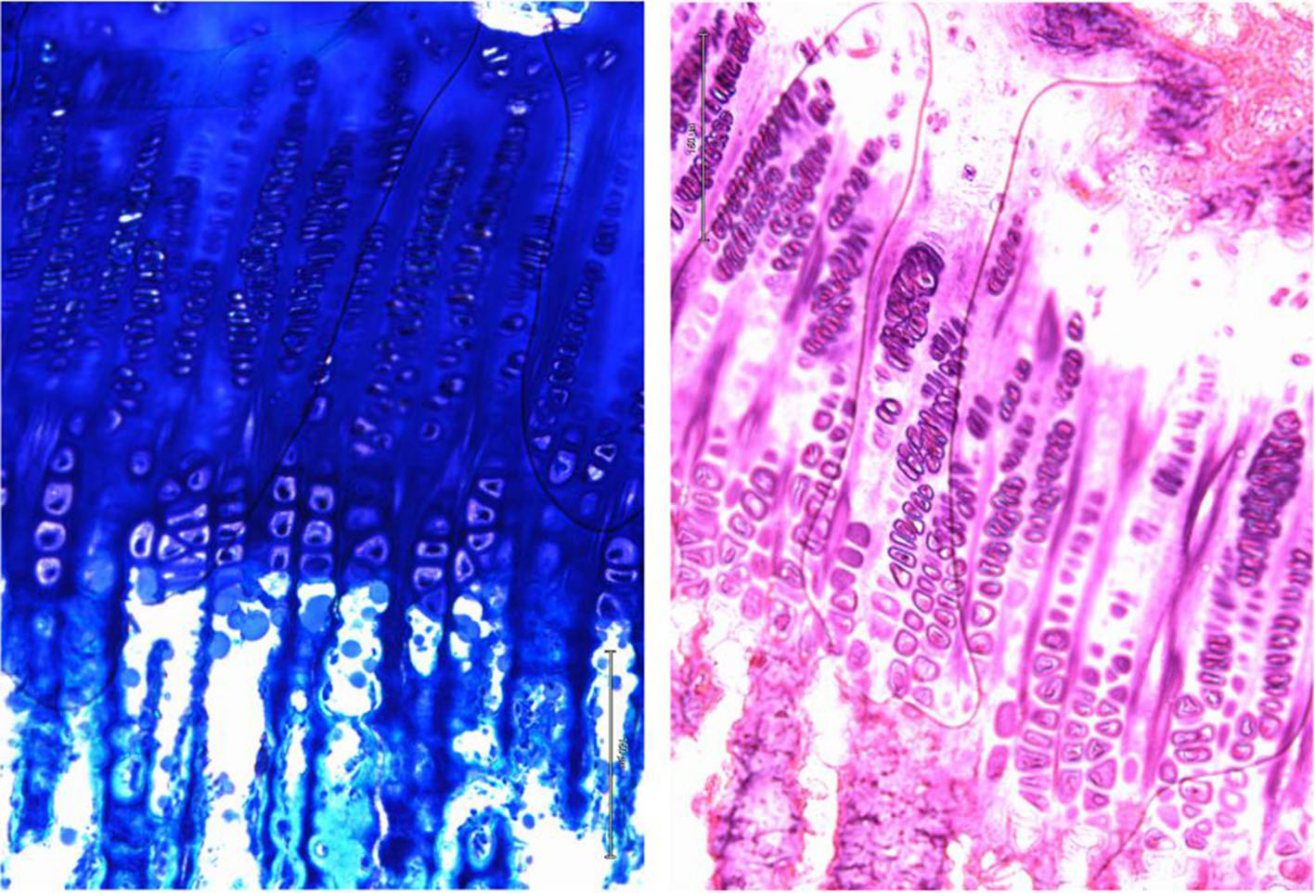
Normal growth plate ×10. Toluidine blue and H&E staining are both useful to describe the growth plate morphology

**Fig. 5:**
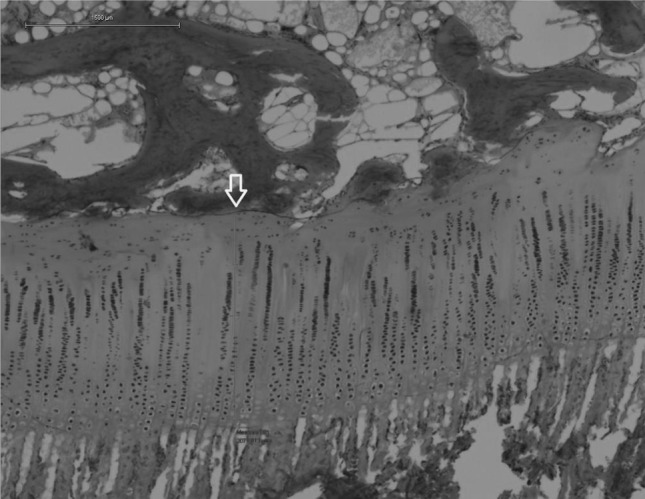
Normal growth plate, ×4. Homogeneous thickness (*arrow*) and column arrangement can be observed

The samples were decalcified and embedded in cold methyl methacrylate (MMA). Coronal 7-µm sections were taken from each block until four sections were obtained where the growth plate was visible. Haematoxylin and eosin (H&E) and toluidine blue (normal pH) were used for staining (Fig. 4). Measurements were done on the H&E-stained samples (Fig. 5).

### Analysis

Two sections with each staining were analyzed from each block (approx. 1 mm distance between) using a computer-assisted microscopy stereology system at 1.25×, 4× and 10× (CAST, Visiopharm, Hoersholm, Denmark). For each sample, the following parameters were considered:

- *Growth plate continuity* The growth plate is seen as a continuous line [8]. After RFA epiphysiodesis, it is expected that the growth plate will be disrupted at the ablation sites.
- *Growth plate thickness* Along a normal growth plate, no thickness variations are expected to be found. Edema and injury can affect the growth plate volume [10]. Changes in growth plate thickness are expected to be found at the ablation sites.
- *Growth plate columnar organization* The growth plate is a columnar-arranged structure [10]. This arrangement is expected to be affected after RFA epiphysiodesis.
- *Possibility of distinguishing organized layers* A sandwich-like arrangement in layers is described in normal growth plates. This arrangement is affected by injury [10]. RFA epiphysiodesis is expected to disarrange the normal organization of the growth plate.
- *Presence or absence of bone bridges* Bone bridge formation can be caused by growth plate injury or fracture [2]. Bone bridges are expected to be evident after RFA epiphysiodesis.

The data obtained from the treated tibiae were compared to those from the non-treated, which were considered as control.

## Results

*Non-treated specimens* Histological sections from controls showed a continuous and organized growth plate. Layers could be identified and columnar organization was observed (Fig. 5). The mean thickness was 625 µm (606–639, SD = 14). No bone bridges were observed. All the control specimens were classified as normal.

### Treated specimens

**Fig. 6:**
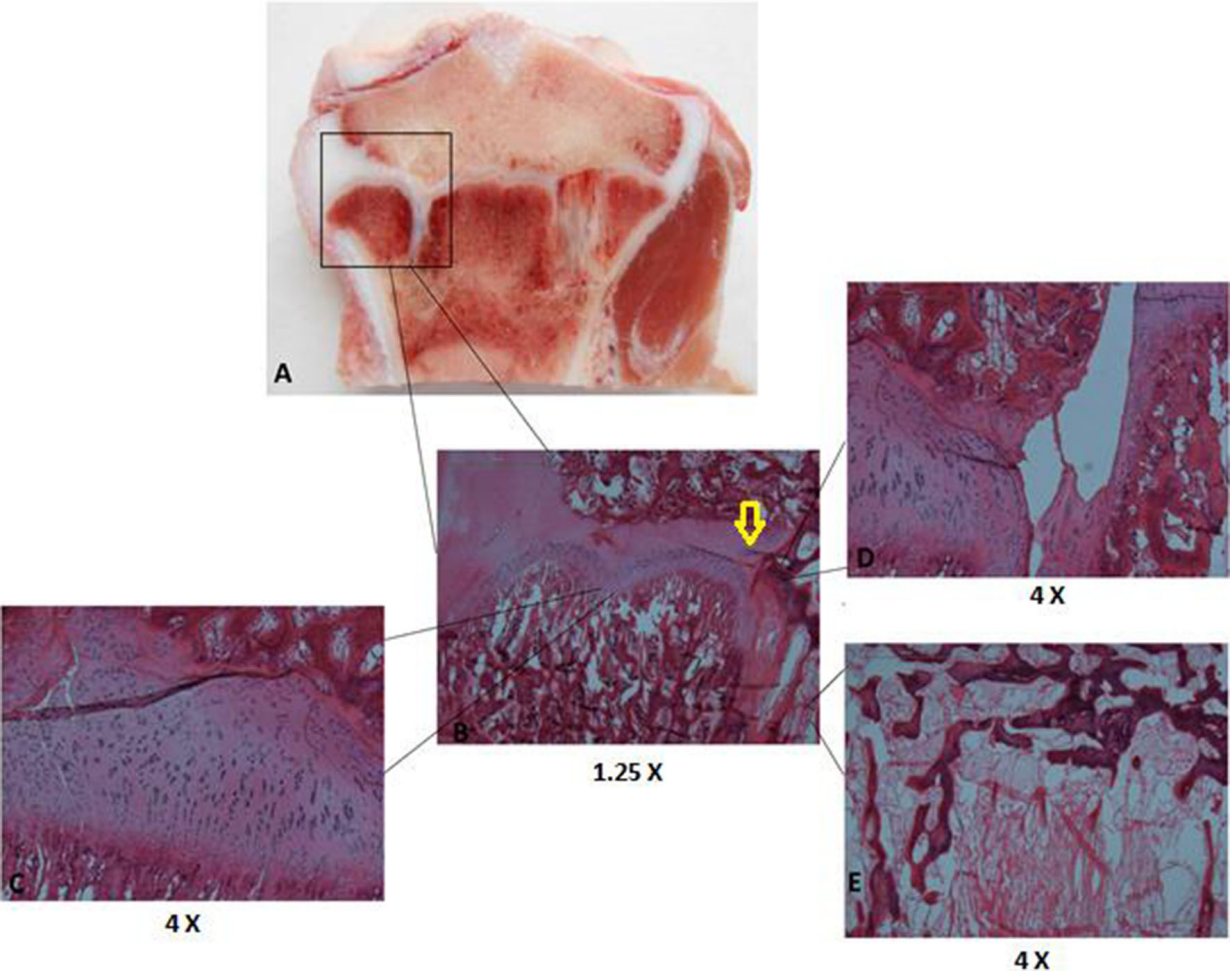
Histology of the ablated growth plate. In the ablated site (*framed*, **a**), the growth plate could be followed until it widened and presented fibrosis (*arrow*, **b**) followed by discontinuity (**d**) and a bone bridge (**e**). In addition, disorganization could be observed along the growth plate (**c**)

*Growth plate continuity* Sections from the treated physis presented a discontinuity of the growth plate (Fig. 6d) at the ablation sites.

*Growth plate thickness* The central part of the physis looked normal. Next to the bone bridge, the physis looked thicker (Fig. 6b). The mean thickness of the treated physis was 820 µm (628–949, SD = 130).

*Growth plate columnar organization* At the ablation sites, it was observed that the normal columnar organization of the growth plate was lost. Torsion and angulation of the columns was found (Fig. 6c).

*Possibility of distinguishing organized layers* At the ablation sites and next to them, disorganized layers in a heterogeneous line were observed (Fig. 6c).

*Presence or absence of bone bridges* Bone bridges were identified in all the treated specimens (Fig. 6e). They were flanked by fibrosis (Fig. 6b).

## Discussion

The mechanism of endochondral bone growth is incompletely understood and continues to be an active and dynamic area of research [11]. To date, the exact post-fractural reactions of the growth plate are poorly understood. Previous studies have indicated that bone bridge formation does not involve endochondral ossification, and also that there is an initial inflammatory response involving infiltration of inflammatory cells at the growth plate injury site [1]. Epiphysiodesis using RFA was reported as successful but histology was not described [8]. Our results are in accordance to previous published studies. In a rabbit model, using percutaneous drilling, Kömür et al. reported, at the treated sites, significant losses of the physis zones in the treated group after the second post-operative week, a normal columnar arrangement at the non-treated physeal regions, and presence of bone bridges [4]. In addition, in a RFA epiphysiodesis study using rabbits, Ghanem et al. reported that treated specimens revealed cellular anarchy, loss of columnar stratification, and loss of the physeal height, while the medial counterpart remained normal [5]. Sgariglia et al. reported that injured physes are characterized by disorganization of the proliferative, pre-hypertropic and hypertrophic zones [12]. Changes in the growth plate can be observed after 7 days of injury. After 14 days they are evident, and after 4 weeks evidence in growth can be observed [2]. Bone bridges observed in our samples may have caused growth arrest; our animals were followed for 12 weeks. Previous histological studies were done in small species (rabbits and rodents), and it is important to emphasize that similar results were observed in our study which used a large species (pig) where the growth plate volume is higher and the bone structure more similar to human [13]. We used H&E and toluidine blue staining which allows an accurate morphological analysis. It has been reported H&E staining is the best for measurements, except when metabolic parameters are studied, which would require special staining [1, 11, 12, 14]. Light microscopy is been reported to be adequate for analyzing growth plate morphology [6]. In conclusion, epiphysiodesis performed using RFA disrupts the growth plate morphology and causes the formation of bone bridges at the ablation sites. The damaged tissue, next to the bone bridges, is characterized by disorganized structures of the physis and presence of fibrosis.
